# Cholinergic Modulation of Narcoleptic Attacks in Double Orexin Receptor Knockout Mice

**DOI:** 10.1371/journal.pone.0018697

**Published:** 2011-04-13

**Authors:** Mike Kalogiannis, Emily Hsu, Jon T. Willie, Richard M. Chemelli, Yaz Y. Kisanuki, Masashi Yanagisawa, Christopher S. Leonard

**Affiliations:** 1 Department of Physiology, New York Medical College, Valhalla, New York, United States of America; 2 Department of Neurosurgery, Washington University, St. Louis, Missouri, United States of America; 3 Howard Hughes Medical Institute and Department of Molecular Genetics, University of Texas Southwestern Medical Center, Dallas, Texas, United States of America; 4 Department of Neurology, Ohio State University, Columbus, Ohio, United States of America; Pennsylvania State University, United States of America

## Abstract

To investigate how cholinergic systems regulate aspects of the sleep disorder narcolepsy, we video-monitored mice lacking both orexin (hypocretin) receptors (double knockout; DKO mice) while pharmacologically altering cholinergic transmission. Spontaneous behavioral arrests in DKO mice were highly similar to those reported in orexin-deficient mice and were never observed in wild-type (WT) mice. A survival analysis revealed that arrest lifetimes were exponentially distributed indicating that random, Markovian processes determine arrest lifetime. Low doses (0.01, 0.03 mg/kg, IP), but not a high dose (0.08 mg/kg, IP) of the cholinesterase inhibitor physostigmine increased the number of arrests but did not alter arrest lifetimes. The muscarinic antagonist atropine (0.5 mg/kg, IP) decreased the number of arrests, also without altering arrest lifetimes. To determine if muscarinic transmission in pontine areas linked to REM sleep control also influences behavioral arrests, we microinjected neostigmine (50 nl, 62.5 µM) or neostigmine + atropine (62.5 µM and 111 µM respectively) into the nucleus pontis oralis and caudalis. Neostigmine increased the number of arrests in DKO mice without altering arrest lifetimes but did not provoke arrests in WT mice. Co-injection of atropine abolished this effect. Collectively, our findings establish that behavioral arrests in DKO mice are similar to those in orexin deficient mice and that arrests have exponentially distributed lifetimes. We also show, for the first time in a rodent narcolepsy model, that cholinergic systems can regulate arrest dynamics. Since perturbations of muscarinic transmission altered arrest frequency but not lifetime, our findings suggest cholinergic systems influence arrest initiation without influencing circuits that determine arrest duration.

## Introduction

Narcolepsy is a human sleep disorder characterized by excessive daytime sleepiness, sleep paralysis, hypnagogic hallucinations and cataplexy – epochs of muscle atonia triggered by strong emotions [1]. Evidence indicates that the disease results from a deficiency in orexin neuropeptides [2], [3]. These neuropeptides (also called the hypocretins) are synthesized by lateral hypothalamic neurons [4], [5] having extensive central projections [6]. Two G-protein coupled orexin receptors (OX1R; OX2R) have been identified [5] and are expressed throughout the brain in a partly overlapping pattern [7], [8]. Studies linking orexin deficiency to human narcolepsy quickly followed upon the discoveries that prepro-peptide null mice have a narcolepsy phenotype [9] and canines, with heritable narcolepsy, have a null mutation in the OX2R [10]. Interestingly, OX2R null mice are less severely affected [11], presumably due to residual OX1R signaling. Preliminary reports indicate that OX1R null mice lack narcolepsy symptoms and do not have cataplexy [12], [13] while double orexin receptor knockout (DKO) mice exhibit narcolepsy with cataplexy [13], [14].

Despite these clues, it remains unclear how the loss of orexin signaling produces narcolepsy. Orexin synaptic targets at the pontomesencephalic junction, including noradrenergic neurons of the locus coeruleus, the serotonergic neurons of the raphe system and the cholinergic neurons of the laterodorsal tegmental (LDT) and pedunculopontine (PPT) nuclei, are candidates for mediating arousal-related functions of orexin [15]. Moreover, these structures have long been thought to function in generating EEG and muscle tone changes associated with REM sleep. Several lines of evidence support a model in which reciprocal synaptic interactions between REM-on cholinergic and REM-off monaminergic neurons form a switching mechanism that determines the onset and duration of REM sleep and its associated signs such as muscle atonia (for review see [16]). Indeed, results from studies in narcoleptic canines indicate an imbalance in brainstem cholinergic and monoaminergic transmission contributes to the expression of cataplexy, as might be expected from this model (for review see [17]). Nevertheless, accumulating evidence from rodent studies [18], [19], [20] has challenged the importance of cholinergic and monoaminergic interactions in the generation of REM-sleep. This work has led to models in which interconnected REM-off and REM-on GABAergic neurons form the core elements of a REM sleep switch. In these models, cholinergic and monoaminergic neurons function outside the switching mechanism, although their roles have not yet been defined (for review see [21], [22]).

In contrast to canine narcolepsy, little is known about the pharmacology of rodent narcolepsy/cataplexy. We therefore characterized for the first time the dynamics of narcoleptic attacks in DKO mice and determined whether altered cholinergic transmission, produced by cholinesterase inhibitors and atropine, affect these dynamics. We found that narcoleptic arrests of DKO mice appear to be a phenocopy of those in orexin deficient mice and that arrest lifetimes follow an exponential distribution. We also found that enhanced cholinergic transmission increased the number but not the lifetime of narcoleptic arrests in DKO mice, but did not produce arrests in wild-type mice. These findings imply that cholinergic outflow in orexin-signaling deficient mice can regulate transitions into, but not out of, the arrest state. This is consistent with cholinergic systems influencing arrest initiation circuitry without being part of the circuits determining arrest duration.

## Methods

### Ethics Statement

All protocols used in this study were reviewed and approved by the Institutional Animal Care and Use Committee of New York Medical College and were compliant with NIH guidelines for ethical treatment of animals.

### Mice

DKO breeder mice from the Yanagisawa laboratory were re-derived by embryo transfer at New York Medical College and the offspring from these re-derived mice were used for this study. DKO mice were originally created by cross breeding heterozygous progeny of homozygous single receptor knockouts (OX1RKO×OX2RKO). The making of OX2RKO mice have been described in detail [11] and the making of OX1RKO mice has been described in a preliminary report [12] and will be described completely in a future publication that will also include polysomnography data from both DKO and OX1RKO mice. Background control WT mice were derived from the WT littermates resulting from heterozygous OX2RKO crosses. Hence both strains of mice used in these experiments had the same mixed C57Bl6×129/SvEv genetic background.

Genotyped male DKO and WT mice aged 12–20 weeks old (28–32 g) were housed individually in rat cages (10.25″W×18.75″D×8″H) equipped with a running wheel with unlimited access to water and standard lab chow. The mice were maintained on a 12∶12 light-dark cycle, with 7:00 AM as the beginning of the light phase. Mice were acclimated to these new home cages for five days before recording began. Cage bedding was replaced once every week after a video recording session. All video recording sessions lasted for three hours and began a half hour prior to lights out, a period when the most number of cataplexy-like attacks were expected [9].

### Video recording and behavioral analysis

Infrared sensitive video cameras (MCM electronics) were mounted onto the top of home cages and video streams from these cameras were digitized simultaneously at 320×240 pixels/frame at 15 frames per second by an Axis 241Q video server (Axis Communication, Lund Sweden) and recorded to a MacBook Pro. SecuritySpy software (Bensoftware) running on the Mac was used to configure the server and to capture, compress (MPEG-4) and time-stamp the video. Behavioral scoring was performed using Annotation (Saysosoft) software. Mice were randomly assigned to one of two observers for scoring. To achieve congruent scoring criteria, a separate cohort of mice were scored by both observers until they were consistent in their criteria prior to working with the mice used for these experiments. Any behavior deemed ambiguous by one observer in the experimental mice was reviewed by both observers to assign a score. Onset and duration data for behavioral events were then imported into custom analysis procedures developed in Igor Pro (Wavemetrics, Lake Oswego, OR, USA) which allowed us to summarize and compare behavioral events between individual mice and across genotypes. Computation of cumulative distributions or event duration and survival curves was done using custom procedures in Igor Pro. Curve fitting was accomplished by fitting survival distributions to built-in Igor Pro functions for a shifted exponential (y(x) = y_0_+Aexp(−(x-x_0_)/tau)) and a power function (y(x) = y_0_+Ax^−alpha^) with x_0_ set to 5 s, and y_0_ constrained to be 0, using their built-in implementation of the Levenberg-Marquardt algorithm. Additional tabulation was done using Excel (Microsoft).

### Systemic injections

To investigate the influence of cholinergic signaling on behavioral arrests, 20 mice (n = 10 DKO; n = 10 WT) received IP injections of either physiological saline (0.9% NaCl) or physostigmine dissolved in saline (0.01, 0.03, and 0.08 mg/kg body weight; Fisher Scientific cat # 50809809) a half hour prior to video recording. Each mouse received a single injection once every 5 days for a total of 4 injections in random order. In a separate series of measurements, seventeen mice (DKO, n = 10, WT, n = 7) received atropine (0.5 mg/kg body weight; Fisher Scientific cat # ICN10081301) or saline injections in random order a half hour prior to recording (one hour before the start of the dark cycle).

### Pontine Microinjections

Twenty mice (DKO, n = 11; WT, n = 9) were each surgically implanted with a guide cannula (PlasticsOne, Roanoke, VA) following a modified protocol previously published [23]. Mice were anesthetized with ketamine (100 mg/kg)/xylazine (3.5 mg/kg) ip and placed in a Kopf (Tujunga, CA, USA) model 962 stereotaxic frame with non-rupture, cup-type ear bars. The skull was exposed and a 26 gauge stainless steel guide cannula (PlasticsOne) was stereotaxically implanted at a depth 2 mm above the pontine reticular nuclei (−4.7 mm posterior and −0.7 mm lateral from bregma). A dummy cannula of the same length was placed into the guide cannula and Cerebond (PlasticsOne) was used to adhere the assembly to the skull. Following a 1 week recovery period, each mouse received an injection once every 4 or 5 days a half hour prior to recording. Recordings began 30 minutes before lights-off and continued for a total of three hours. On the day of injection, the dummy cannula was replaced with a 32 gauge injection cannula. The injection cannula extended 2 mm past the guide cannula to reach the nucleus pontis oralis (PnO) or caudalis (PnC). A total of 50 nl was delivered over a 6 minute period via a microinjector (IM-1, Narashige, Japan) and a 1 µl syringe (series 7000, Hamilton, Reno NV). The internal cannula was kept in place for 1 minute post-injection to allow additional time for diffusion from the injection site. Injections consisted of either ACSF (artificial cerebral spinal fluid; NaCl (124 mM), KCl (5 mM), NaH_2_PO_4_ (1.2 mM), CaCl_2_ (2.7 mM), MgSO_4_ (1.2 mM), NaHCO_3_ (26 mM) and D-Glucose (10 mM)), neostigmine dissolved in ACSF (Fisher Scientific cat # 338440050, 62.5 µM, 0.95 ng) or neostigmine and atropine dissolved in ACSF (62.5 µM, 0.95 ng neostigmine; and 111 µM, 3.8 ng atropine) were given in a random order a half hour prior to the recording session. Neostigmine, like physostigmine, is a cholinesterase inhibitor, but does not cross the blood brain barrier. Coleman et. al. found that a 50 nl microinjection of 0.88 mM neostigmine into the PnO induced REM-like state and decreased REM sleep latency in C57Bl6 mice [23]. In preliminary studies, we found that such high concentrations of neostigmine greatly increased the amount of time the mice spent in the inactive state. We therefore reduced the concentration to levels that did not produce obvious effects on general behavior.

### Histology

A day after the last injection, the mice were perfused through the heart with saline (40 mls) followed by 4% paraformaldehyde (PFA; 1000 mls). The brains were then removed and submerged in 4% PFA overnight followed by 30% sucrose. Brains were sectioned on a cryostat at 50 µm, were processed with cresyl violet staining and examined by brightfield microscopy (Olympus BX60) to verify cannula placements. Location of the injections sites were then plotted onto standard drawings from an atlas [24].

### Statistical Analyses

All data is shown as mean ± standard error of the mean. Differences between means were determined by two-way ANOVAs with mouse and treatment as factors using DataDesk 6.2 software (Data Description, Ithaca, New York) and protected Fischer's Least Square Difference post-hoc testing where significance was taken as p<0.05 when multiple means were compared. Additional non-parametric Kruskal-Wallis post-hoc testing was performed using NCSS software (NCSS, Kaysville, Utah) which confirmed the results from the parametric post-hoc tests. Bout duration distributions were compared using the Kolmogorov-Smirnov (K-S) test implemented in Igor Pro software with significance set at p<0.05.

## Results

**Figure 1:**
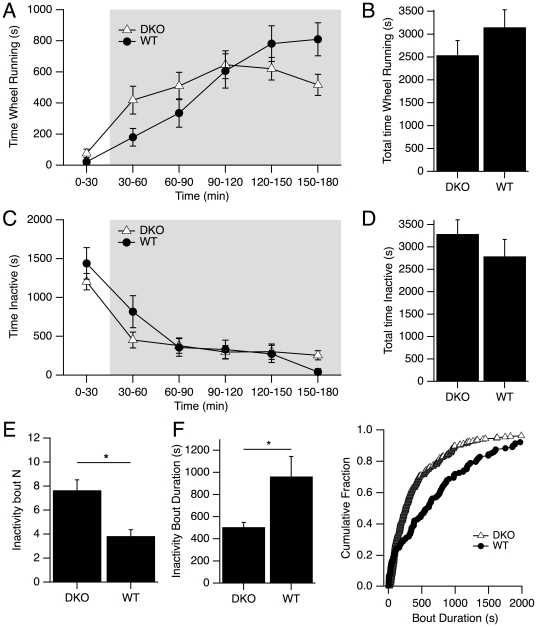
Wheel running and Inactivity in DKO and WT mice. (A) Average (± SEM) time spent wheel running per mouse measured in 30-minute bins across the entire recording session for DKO and WT mice. (B) The average (± SEM) time per animal over the three-hour recording period spent wheel running. (C) Average (± SEM) time spent “inactive per mouse measured in 30-minute bins across the entire recording session. (D) The average (± SEM) time per mouse spent inactive for the three-hour recording session. Both the temporal pattern and amount of wheel running and inactivity were similar in DKO and WT mice (E) In contrast, there were many more bouts of inactivity in DKO mice (mean ± SEM). In E and F * denotes p<0.05 by a one-way ANOVA. (F, *left*) Moreover, inactivity bouts duration were shorter (mean ± SEM) in DKO mice. (F, *right*) Cumulative distributions of inactivity bout duration. Bouts were significantly shorter in DKO mice (K-S test p<0.05).

In order to gauge the overall level of mouse activity during the recording period we used two measures. The first was the amount of time the mice spent interacting with the running wheel. As expected, both WT and DKO mice exhibited an increased wheel activity in the dark period (Fig. 1A). We found that there was no significant difference between genotypes in the average time mice spent interacting with the wheel over the three hour recording time (WT: 3151.0±382.8 s; DKO: 2539.7±320.3 s; p>0.05; Fig. 1B). In addition, we found no difference between genotypes in the distribution of wheel running bout durations or the mean bout duration (mean bout duration WT: 90.9±14.4 s vs. DKO: 84.3±10.6 s; p>0.05). Of course, there may have been differences in speed and distance covered, as reported for the orexin ligand knockout [25] but we only measured the time interacting with the wheel. It therefore seemed that the DKO mice were as interested by the wheel as were the WT mice.

As a second indicator of activity, we measured the time spent in behaviors we classified as “inactive”. Inactive behaviors were epochs of prolonged cessation of movement that were most commonly preceded by nesting behaviors. Nesting behaviors were recognized as repeated circular movement of the mouse near the sides or corner of the cage and/or movement of bedding to form a nest. During periods of inactivity, the mouse was often curled, minimizing the body volume to surface ratio. Most inactivity bouts occurred in corners, although some were observed along the sides of the cage and even under the wheel. Since these behaviors undoubtedly included sleep bouts, it was not surprising that they had a roughly opposite temporal pattern to wheel running in both WT and DKO mice (Fig. 1C). Moreover, the total time spent in this behavior was not different between genotypes (WT: 2791.0±376.8 s/mouse; DKO: 3287.8±317.1 s/mouse; p>0.05; Fig. 1D). Interestingly, DKOs had about twice as many bouts of inactivity as WTs (WT: 3.8±0.5/mouse vs. DKO: 7.7±0.9/mouse; p<0.05; Fig. 1E) and the average bout duration was about half of that measured in WT mice (WT: 963.7±180.5 s, DKO: 506.10±43.0 s; p<0.05; Fig. 1F, left). As can be seen in the cumulative inactivity bout duration (Fig. 1F, right), the range of inactivity bout durations was similar for DKO and WT mice, but there were many more bouts of shorter duration in the DKOs (K-S test, p<0.05). Since these bouts were largely made up of time asleep, these data indicate that DKO mice have an inability to maintain consolidated rest behavior, presumably as a result of the sleep and wake fragmentation which has previously been extensively documented in orexin ligand knockout and orexin-ataxin-3 transgenic mice [26], [27] and noted for DKO mice [13].

### DKO mice exhibited spontaneous behavioral arrests

A striking feature of the DKO mice was the occurrence of spontaneous behavioral arrests that appeared similar to those described for mice lacking the prepro-orexin peptide [9] or orexin neurons [26]. These arrests typically appeared as a collapse in posture with the head and tail laying on the bedding and with the body elongated or covering the limbs. These arrests were generally quite obvious since they were preceded by purposeful motor activity and were sudden in their onset and offset (see video S1). During these arrests, the mice often showed brief phasic movements of the head, tail or limbs. Indeed, a quivering or low amplitude jerky rocking motion was often observed during the attack (see video S2) and often occurred just before resumption of purposeful behavior. Such behaviors were never observed in WT mice. To score these events as full arrests, we required twenty seconds of purposeful behavior prior to and following, at least a temporary cessation of voluntary movement (>5 s). As defined, these arrests likely included both the abrupt and the more gradual onset arrests described by Willie et al. (2003) in OX2R−/− mice and orexin ligand KO mice.

In addition to full arrests, we sometimes observed episodes where movements ceased only briefly (<5 seconds) and head movements or attempted ambulation continued throughout the attack (see video S3). These events were reminiscent of cataplectic attacks that did not proceed to completion in narcoleptic canines and in humans where only some muscle groups become affected [28]. These incomplete attacks also sometimes preceded full arrests and were often initially apparent as a change in gait, which was then followed by a full loss of body support (see video S4). We found that approximately 45% of these “partial” arrests went on to full arrests but these partial arrests were not obligate precursors of full arrests since only about 20% of full arrests were preceded by such behaviors. These appeared similar to reported “gait disturbances” described in the orexin knockouts [9] which would either resolve or progress to a complete arrest. Indeed, a comparable number (27%) of arrests were preceded by these gait disturbances in orexin knockouts.

**Figure 2:**
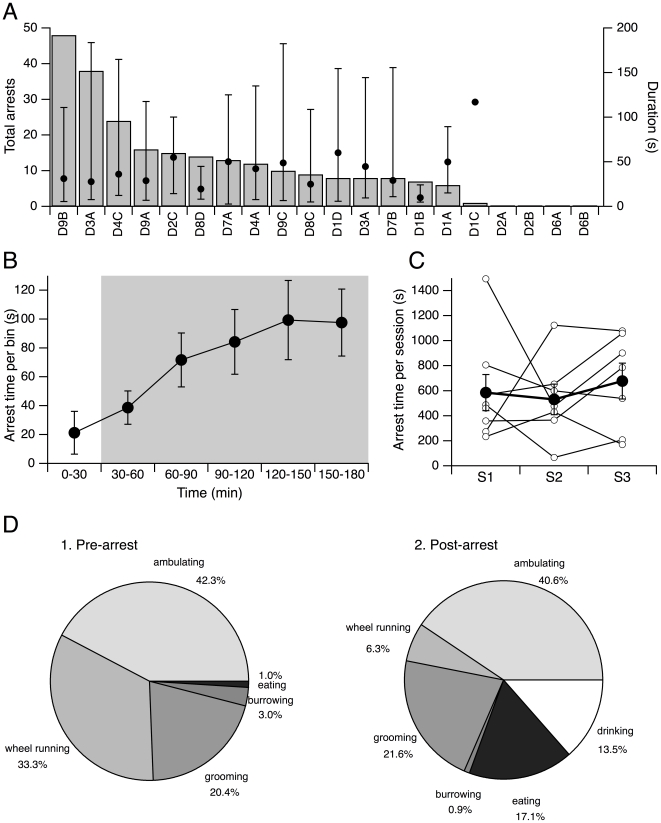
Behavioral arrests in DKO mice. (A) Behavioral arrests in individual DKO mice. *Left scale* shows the total number of behavioral arrests in the three hour recording period by mouse (bars). *Right scale* shows the average duration of all recorded behavioral arrests (s; filled symbol) with the shortest and longest bouts indicated by the whiskers. (B) Mean (±SEM) total time per animal spent in behavioral arrest per 30 min bin shows that time spent in arrests increased substantially after the lights went out. (C) Total time per session for each mouse was variable (open symbols) but the mean (±SEM) time per mouse was stable across sessions (filled symbols). (D) Distribution of DKO behaviors prior to (1) and after (2) behavioral arrests.

As also noted in the original report of behavioral arrests in orexin ligand knockout mice [9], we found that the number of arrests varied greatly among DKO mice. By counting all arrests in the three hour recording period for twenty DKO mice, we found that four mice did not exhibit any arrests, while three exhibited over twenty. Seven mice had between ten and twenty arrests while five mice experienced fewer than ten (Fig. 2A). In addition, for any given mouse, the duration of each arrest varied considerably. This is indicated in Fig. 2A by the range of arrest durations which is plotted along with the mean arrest duration for each mouse. The mean arrest duration across mice was 42.2±6 s (n = 16) which is comparable to the mean arrest duration in ataxin-3 mice and ligand knockout mice which ranged from 41 s to 66 s [9], [11], [26], [27].

We also found that the behavioral arrests were not uniformly distributed across our recording session (Fig. 2B). Few arrests occurred in the initial 30 minutes before the room lights went out and arrest frequency increased after light offset. In the first 30 minutes (before lights out), there was an average of only 0.7 arrests/mouse which occupied 21.3±14.8 s/mouse (n = 20). This increased to an average maximum of 3 arrests/mouse in the 30 minutes beginning two hours after recording onset (1.5 hours after lights out) which occupied 99.3±27.4 s/mouse (n = 20) of this period. On average, the time spent in arrests was well correlated with the time spent wheel running (Pearson's r = 0.97 for mean time in arrest vs. mean time wheel running) and is consistent with the idea that access to the running wheel promotes behavioral arrests in narcoleptic rodents [25].

To determine how variable behavioral arrests were across recording sessions, we recorded eight mice in three successive recording sessions separated by several days (Fig. 2 C). While there was great variability between mice within each recording session, and there was considerable variability for each mouse between sessions, the group average was not statistically different across sessions (p = 0.72, repeated measures ANOVA). Thus, the group average of total time in arrest was a stable measure of behavioral arrests across recording sessions.

To address the behavioral context in which arrests occurred, we examined the behaviors in the twenty seconds before and after arrests for all DKO mice used in this study. Arrests in DKO mice were most often preceded by ambulation (42.3%). This was followed in frequency by wheel running (33.3%) and grooming (20.4%) while only a small fraction of the arrests were preceded by eating or burrowing (total of 4%). Arrests typically ended by an abrupt resumption of activity. The most common of these activities was ambulating (40.6%), followed by grooming (21.6%), eating or drinking (17.1%; 13.5% respectively), wheel running (6.3%) and burrowing (0.9%). Interestingly, these DKO mice were much more likely to eat or drink just after an arrest than just prior to one (Fig. 2D1, 2). These behaviors preceding arrests in DKO mice occurred with similar frequency prior to arrests in ligand knockouts and ataxin-3 mice [9], [26]. For example, 41% of the arrests in ligand knockout mice and 56% of arrests in the ataxin-3 mice were preceded by periods of ambulation. Similarly, 23% of the attacks in ligand knockouts and 30.3% of attacks in the ataxin-3 mice occurred after grooming. Collectively, these data indicate that DKO mice display a narcolepsy-like phenotype that is similar to that reported for mice lacking orexin peptides.

### Behavioral arrest lifetimes are exponentially distributed

**Figure 3:**
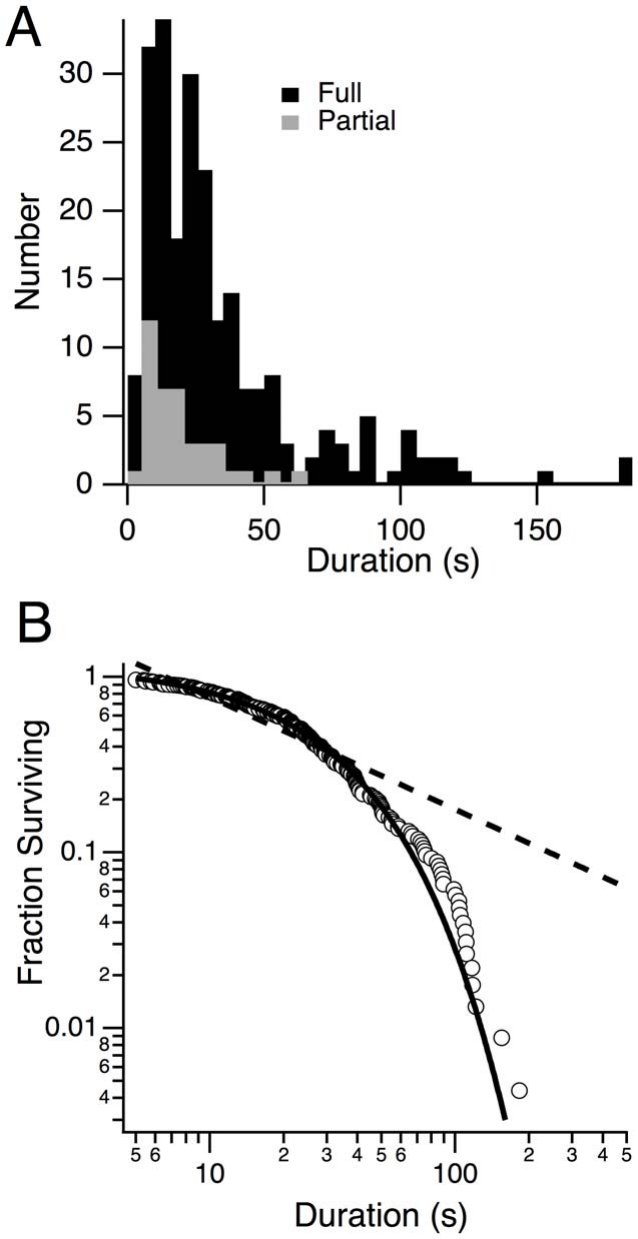
Behavioral arrest duration in DKO mice are well fit by an exponential distribution. (A) Distributions of full (black bars) and partial (grey bars) arrest bout durations. (B) Survival analysis of full arrest bouts. Fraction of bouts longer than each duration are plotted on a double log scale (open symbols). The best fit exponential (solid line) and power functions (dashed line) are superimposed. Arrest lifetimes are poorly fit by a power function but very well fit by the exponential. A small fraction (<13%) of arrests are longer than expected for the best fit exponential relation and clustered around 100 s.

To gain insight into the processes governing expression of behavioral arrests, we examined the distribution of arrest durations. This is illustrated for full arrests in Figure 3A with the distribution of just partial arrests superimposed. Partial arrests made up only a small fraction of all arrests and they were on average, shorter in duration than full arrests. The mean duration for all arrests was 30.9±1.8 s (n = 267 from 16 mice) compared to 33.1±2.2 s for full arrests (n = 227) and 18.7±2.2 s (n = 40) for partial arrests. It can also be seen that the arrest duration distribution was approximately exponential in shape. To test this, we conducted a survival analysis of full arrests (Fig. 3B) where the fraction of full arrests (n = 227) lasting longer than each indicated duration is plotted on log-log scales. Since recent studies have found that the lifetimes of waking episodes are distributed as a species-invariant power law and lifetimes of non-REM sleep are distributed exponentially [29], [30], [31], we compared full behavioral arrest lifetimes to both power and exponential distributions. If arrest durations were distributed as a power function (i.e. arrest lifetime∼t^−alpha^) they would decay linearly on this graph as indicated by the best fit power function (dashed line; alpha = 0.64). Clearly, survival of arrest lifetimes decayed faster and were well fit by an exponential distribution (arrest lifetime∼e^−t/tau^) especially for durations less than 60 seconds as indicated by the solid line. The characteristic time of this best-fit exponential (tau = 26.7 s), which is also the mean of an exponential distribution, agreed well with the actual mean of all full arrests (33.1±2.1 s, n = 227). This finding indicates that once DKO mice enter a full behavioral arrest, the time spent in that arrest is well described by an exponential distribution. At the long end of the distribution (>60 sec), which comprised <13% of the arrests, there was a cluster of arrests with durations around at ∼100 seconds that were longer than expected for an exponential distribution. These may reflect rare arrests that are governed by different kinetics or arrests that transition into a sleep state. For example, REM sleep lifetimes are exponentially distributed for short durations but have another mode around 100 s [32], [33].

### Low doses of physostigmine acutely exacerbate narcolepsy in DKO mice within the first hour of recording

To investigate the potential role of cholinergic systems in regulating behavioral arrests, we first studied the effect of altering cholinergic transmission systemically with IP injections of physostigmine or atropine. We then studied the effects of altering cholinergic transmission locally in the pontine reticular formation by microinjecting neostigmine or neostigmine and atropine. In each of these experiments we focused on *full* behavioral arrests, which we considered to be manifestation of narcolepsy.

**Figure 4:**
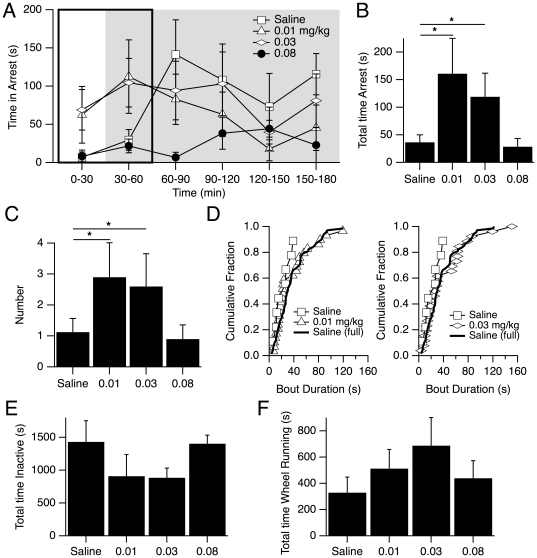
The number of behavioral arrests in DKO mice increased following low doses of systemic physostigmine. (A) Mean (± SEM) time per mouse spent in full behavioral arrests are plotted in 30 min bins across the entire recording period following IP injections of saline and three concentrations of physostigmine (0.01, 0.03 and 0.08 mg/kg). Following the two lower doses, time in arrest was elevated in the first recording hour (boxed area). The grey background indicates dark period of the recording. (B) Mean (± SEM) time in full behavioral arrest in the first recording hour following each dose. Time spent in arrest differed significantly across doses (p<0.05, 2-way ANOVA) and was elevated ∼3-fold following the two lowest physostigmine doses but was not elevated following the highest dose. (C) Mean (± SEM) number of full behavioral arrests per animal in the first recording hour varied by dose (p<0.05, 2-way ANOVA). The two lower doses increased the number of full behavioral arrests compared to saline. * denotes p<0.05 by post-hoc testing for B and C. (D) Cumulative distributions of arrest bout duration over the first recording hour. Distributions following saline vs. 0.01 mg/kg physostigmine (left; open symbols) and saline vs. 0.03 mg/kg physostigmine (right; open symbols) were compared using the Kolmogorov-Smirnoff test. Arrest bout durations following physostigmine were not different than those following saline although some longer bouts were present. The cumulative distribution of all bouts following saline is also shown (dark line) and it superimposes over the distribution following physostigmine. (E) Mean time spent in the inactive state in the first hour of recording following saline and each dose of physostigmine. (F) Mean time spent wheel running in the first hour of recording following saline and each dose of physostigmine.

The cholinesterase inhibitor physostigmine was delivered IP at three doses (0.01, 0.03, 0.08 mg/kg) to twenty mice (DKO, n = 10; WT, n = 10) one half hour prior to video recording (one hour prior to dark phase). We found that the lower two doses of physostigmine produced an increase in the time spent in full arrest within the first hour of recording (Fig. 4A,B). During this period, the mean time spent in arrest per mouse differed significantly across doses (p<0.05, 2-way ANOVA). It increased from 36.5±13.5 s following saline injections to 160.9±63.9 s (0.01 mg/kg; p<0.05) and to 118.9±42.6 s (0.03 mg/kg, p<0.05) following physostigmine injections (Fig. 4B). Consistent with the time course of physostigmine actions [34], [35], which was expected to elevate brain ACh levels for ∼1 hour [36], there was no difference in the time spent in arrest at later recording times. Strikingly, at the highest dose (0.08 mg/kg) the early increase in the time spent in arrest was absent (28.5±14.8 s, not different from Saline, p>0.05; Fig. 4A).

The increase in the time spent in arrest produced by low doses of physostigmine could be due to an increase in the number and/or duration of the arrests. The number of arrests differed significantly by dose (p<0.05, 2-way ANOVA) and were significantly increased compared to saline at the two lower doses (p<0.05; Saline: 1.1±0.4, Physo 0.01 mg/kg: 2.9±1.1; Physo 0.03 mg/kg: 2.6±1.1) but not at the highest dose (Physo 0.08 mg/kg: 0.9±0.5, p>0.05; Fig. 4C). The mean arrest bout duration also differed by dose (p<0.05, 2-way ANOVA; Saline: 25.2 s±4.7; Physo 0.01 mg/kg: 40.2 s±5.5; Physo 0.03 mg/kg: 51.2 s±11.9; Physo 0.08 mg/kg: 18.5 s±2.5) although post-hoc comparisons were significant only between the 0.03 mg/kg dose and the saline and 0.08 mg/kg doses. Interestingly, the distributions of bout durations were not significantly different (K-S test; p>0.05; Fig. 4D) even though there were some longer bouts present following low-doses of physostigmine. In fact, the distribution of bout durations in the first hour following low doses of physostigmine completely overlapped with the distribution of arrest durations from the whole recording period following saline injections, which also included some long bouts (thick line in Fig. 4D). This indicates that following physostigmine injection, the appearance of a few longer arrest bouts, which are rarer than short bouts, would be expected simply because there were more arrests. Collectively, these data suggest that low doses of physostigmine increase the likelihood of full arrests without altering arrest dynamics.

To determine if these doses of physostigmine altered behavioral arrests due to general changes in arousal, we also examined the effects of physostimine on wheel running and inactivity (Fig. 4E,F). We found that the time spent in these activities was not significantly different across drug treatment conditions by 2 way ANOVA with condition and mouse as factors (p = 0.31, wheel running; p = 0.20, inactivity). This indicates that these concentrations of physostigmine alter expression of arrests without producing detectable changes in these measures of arousal or motor activity.

Importantly, none of these physostigmine doses produced behavioral arrests in WT mice. In some cases, it produced side effects such as body tremors or spasms, however these events were rare, brief and observed in both WT and DKO mice. None of these events resembled the behavioral arrests observed in the DKO mice. Thus, we conclude that physostigmine and the resulting enhancement of cholinergic transmission promotes narcoleptic attacks in DKO mice and that the absence of orexin signaling is necessary for this effect.

### Atropine significantly attenuates behavioral arrests in DKO mice

**Figure 5:**
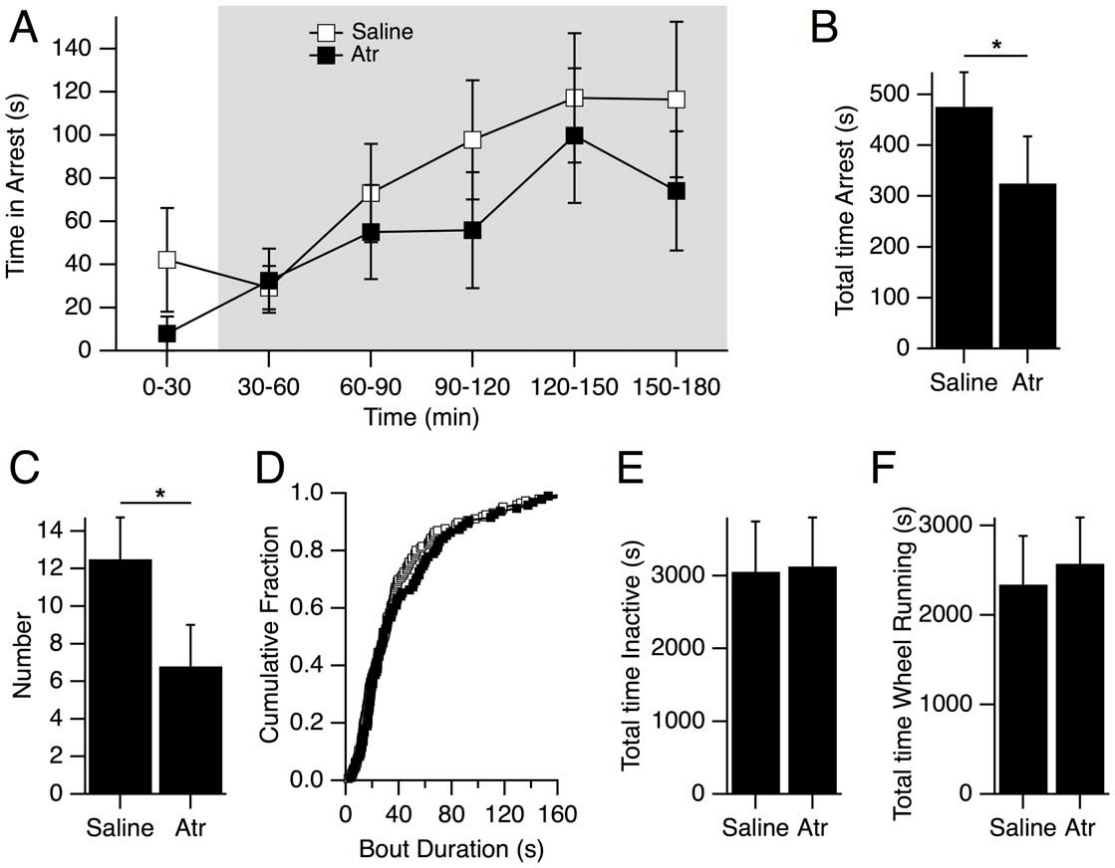
The number of behavioral arrests in DKO mice decreased following systemic atropine. (A) Mean (± SEM) time per mouse spent in full behavioral arrests are plotted in 30 min bins across the entire recording period following IP injections of saline (open symbols) and atropine (0.5 mg/kg; filled symbols). Grey area indicates dark phase. (B) Mean (± SEM) time per mouse spent in arrest over the entire recording following saline and atropine. The time spent in arrest was significantly reduced following atropine. (C) Mean (± SEM) number of arrests per mouse over the entire recording period following saline and atropine. Number of behavioral arrests were also significantly decreased. In B and C * denotes p<0.05 with two-way ANOVAs. (D) Cumulative distributions of arrest durations. Distributions following saline (open symbols) vs. atropine (closed symbols) were compared using the Kolmogorov-Smirnoff test and were not different. (E) Mean (± SEM) time spent in the inactive state was not different following saline and atropine. (F) Mean (± SEM) time spent wheel running was also not different following saline and atropine.

To further test the involvement of cholinergic mechanisms, we administered the muscarinic receptor antagonist, atropine to seventeen mice (DKO, n = 10, WT, n = 7) by IP injection (0.5 mg/kg body weight) thirty minutes prior to video recording. Atropine was expected to reach its highest plasma concentration within two hours after injection [37] and has a four hour half-life [35]. We found that this dose decreased the number of full arrests compared to saline injection. Eight out of the ten DKO mice tested exhibited fewer or no behavioral arrests following injection of atropine compared to saline and the average time in arrest appeared lower during the recording session (Fig. 5A). Across the entire recording session, DKO mice receiving atropine injections spent significantly less time in full arrests (Saline: 475.9±67.9 s, Atropine: 325.1±92.7 s, p<0.05, 2-way ANOVA; Fig. 5B). This decrease was attributable to significantly fewer behavioral arrests (Saline: 12.5±2.2, Atropine: 6.8±2.2, p<0.05, 2-way ANOVA; Fig. 5C) rather than a change in the mean duration of these arrests (Saline: 44.4±7.1 s, Atropine: 54.5±10.5, p>0.05, 2-way ANOVA). In fact, the full arrest bout duration distribution following saline and atropine completely overlapped (KS-test p>0.05; Fig. 5D). Thus, atropine, like IP physostigmine, altered the time spent in full arrests but did not change the duration of arrests once they occurred.

To determine if this effect of atropine was specific for behavioral arrests, we examined the wheel running and inactivity behavior following injections of atropine and saline. We found that neither the number, time, nor bout duration of these activities were altered following atropine (p>0.05, 2 way ANOVA for each), even though the number of behavioral arrests was reduced (Fig. 5E, F). Thus, the effect of this dose of atropine on behavioral arrests did not appear to result from overt changes in overall mouse activity.

Collectively, these data support the idea that muscarinic transmission promotes the expression of behavioral arrests in mice deficient in orexin signaling, but not in WT mice. Moreover, since the number of arrests was altered without affecting arrest durations, these data suggest that cholinergic mechanisms are not involved with establishing the dynamics of the arrests but play a role in regulating their expression. These findings agree well with atropine effects on spontaneous behavioral arrests in orexin ligand KO mice studied under a food shift paradigm (Willie, 2005). In that paradigm, ligand KO mice had a higher rate of arrests than observed for DKO mice studied here in their home cages. Nevertheless, atropine (0.5 mg/kg; IP) decreased the rate of attacks in ligand KO mice to ∼15% of the rate observed following vehicle injection.

### Pontine microinjections of neostigmine increased the frequency of full behavioral arrests

**Figure 6:**
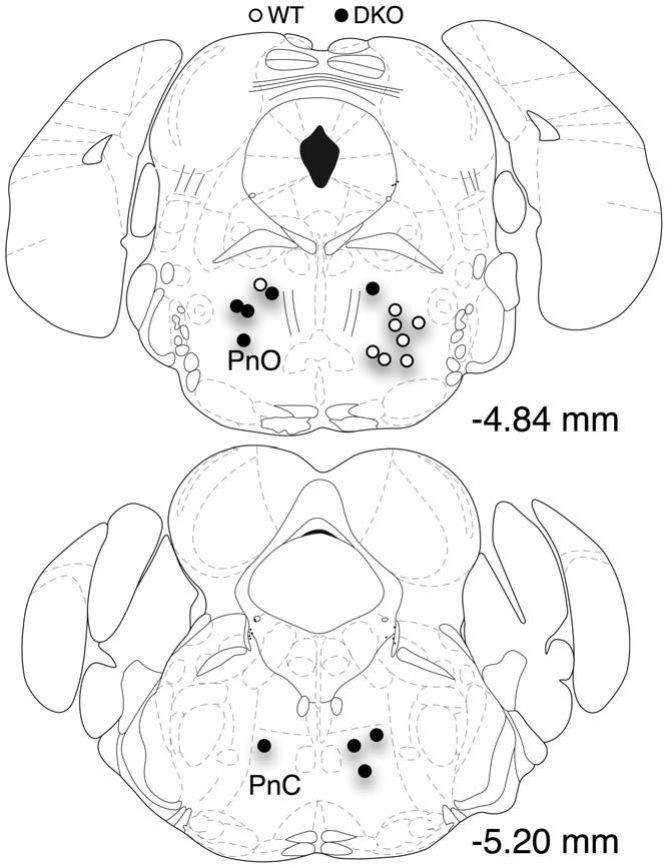
Locations of neostigmine and saline microinjection sites in the pontine reticular formation. Injection sites ranged from −4.36 mm to −5.20 mm from Bregma and were recovered in the PnO or PnC. Open symbols are the sites from WT mice and closed symbols are the sites from DKOs. See text for further explanation.

In light of the evidence linking cholinergic transmission in the pontine reticular formation to the generation of REM sleep signs and canine cataplexy, we sought to determine whether promoting cholinergic transmission in this region influences mouse narcolepsy. We targeted the PnO or PnC in WT (n = 9) and DKO (n = 11) mice for microinjection (50 nl) of either ACSF, neostigmine (62.5 µM, 0.95 ng) or a mixture of neostigmine (62.5 µM, 0.95 ng) & atropine (111 µM, 3.8 ng) and then scored their behavior. Injections sites for three mice (2 DKO; 1 WT) were outside these nuclei so we excluded their data from further analysis. Figure 6 illustrates the injection site locations for the eight WT and nine DKO mice used for analysis. Injection sites were found over a ∼1 mm rostral to caudal extent between −4.36 mm and −5.34 mm caudal to bregma and were plotted onto two sections demarking the midsections of the rostral and caudal halves of this range. Injection sites in the DKO mice (filled circles) were located in both the PnO and PnC while injection sites in the WT mice (open circles) were located in the PnO.

Since we were concerned that the stress of the implants or microinjection might influence the pattern of arrests, we first compared recordings made prior to implantation with recordings made following ACSF microinjections. This revealed that the same DKO mice had fewer arrests after implantation and microinjection. The mean number of arrests/mouse measured in three hours was reduced from 12.5±2.3 prior to implantation to 0.6±0.3 after implantation and microinjections of ACSF (p<0.05). The time spent in arrest was reduced from an average of 534.0±90.7 s to 37.5±18.9 s (p<0.05). There was, however, no significant difference in the distribution of arrest bout durations (K-S test p>0.05), suggesting that the implantation/injection mainly reduce the expression of behavioral arrests but did not influence their dynamics once initiated.

**Figure 7:**
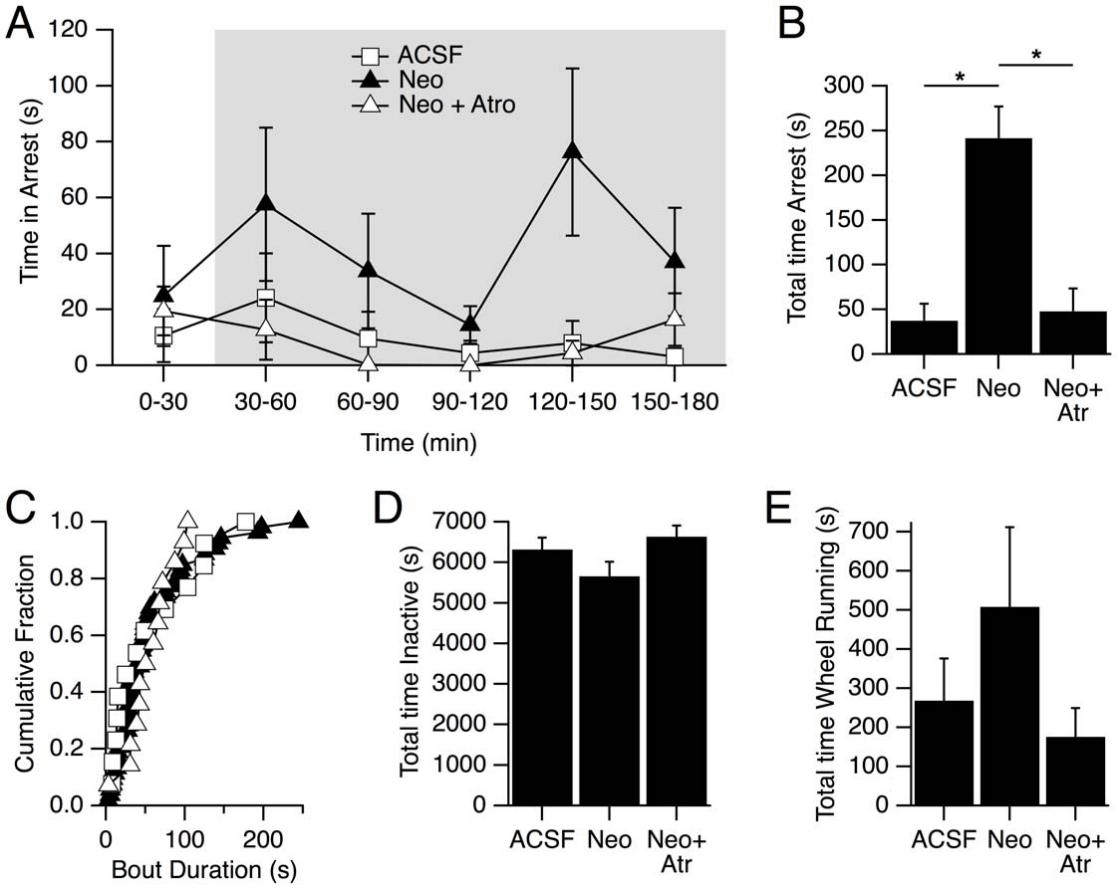
Neostigmine microinjection sites into the PnO/PnC increased behavioral arrests in DKO but not WT mice. (A). Mean (± SEM) time per mouse spent in full behavioral arrests are plotted in 30 min bins across the entire recording period following PnO/PnC microinjections of ACSF (open squares), neostigmine (closed triangles) and neostigmine+atropine (open triangles). Grey area indicates dark phase. (B) Mean (± SEM) time per mouse spent in arrest over the entire recording following ACSF, neostigmine and neostigmine+atropine. Neostigmine increased the time spend in arrest compared to ACSF and neostigmine+atropine, * denotes p<0.05 based on post-hoc testing following a significant two-way ANOVA (p<0.0002). The difference between ACSF and neostigmine+atropine was not significant (p>0.05). (C) Cumulative distributions of arrest bout duration over the entire of recording. Distributions following ACSF, neostigmine or neostigmine+atropine were not different as compared using the Kolmogorov-Smirnoff statistics. (D) Mean (± SEM) time spent in the inactive state. (E) Mean (± SEM) time spent wheel running. Values in D and E were not statistically different across microinjection conditions (p>0.05, 2-way ANOVAs).

We next examined the effects of neostigmine microinjections. We found that 7 out of the 9 DKO mice exhibited more arrests after neostigmine with the average number of arrests increasing more than 4-fold (ACSF: 0.6±0.3, Neo: 4.1±1.0, p<<0.05, 2-way ANOVA) over the three hour recoding period. This resulted in a large increase in the total time spent in full behavioral arrests (ACSF: 37.5±18.9 s, Neo: 241.7±35.8 s, p<0.05, 2-way ANOVA; Fig. 7A,B). However, neither the mean bout duration was altered (p>0.05 2-way ANOVA), nor was the distribution of arrest durations (Fig. 7C) altered, even though the number of arrests was increased (K-S test P>0.05).

To determine if the neostigmine effect on behavioral arrests depends on muscarinic receptor activation, we microinjected a solution of neostigmine with atropine, in order to concurrently block muscarinic receptors. Under these conditions, the number of arrests was not different from the number following microinjections of ACSF alone (Neo+Atr: 0.9±0.4, p>0.05). Moreover, the total time spent in arrest (Neo+Atr: 48.1±25.6 s) was also not different (Fig. 7A,B) but was significantly less than that following Neo alone (p<0.05; Fig. 7B). Despite this, the distribution of arrest durations was not significantly different from that following ACSF or Neo alone (Fig. 7C; K-S test p>0.05).

In contrast to the effect in DKO mice, pontine microinjections of neostigmine into WT mice never produced behavioral arrests or behaviors resembling arrests, indicating that the ability of this dose of neostigmine to promote behavioral arrests required the absence of orexin signaling.

Given the preponderance of rostral injection sites in the WT mice, we compared the effectiveness of neostigmine at rostral and caudal microinjection sites in DKO mice to see if injection location could account for the absence of arrests in WT mice following microinjections. We found that the total time spent in arrest was highly dependent on the injection condition but was not dependent on injection location (PnO or PnC; 2 way ANOVA, drug: p<<0.05; location: p = 0.69). Neostigmine microinjections into the PnC increased the mean time in arrest from 29.5±24.9 s (ACSF) to 229.5±48.0 s (Neo; p<0.05, n = 4) while microinjection of Neo+Atr did not significantly increase the mean time spent in arrest (90.0±52.3 s; p = 0.35, n = 4). Similarly, neostigmine microinjections into the PnO increased the mean time in arrest from 43.8±29.8 s (ACSF) to 251.4±55.2 s (Neo; p<0.05, n = 5) while microinjection of Neo+Atr did not significantly increase the mean time spent in arrest (14.6±9.1 s; p = 0.58, n = 5). Thus, in DKO mice, neostigmine injections into the PnO region were as effective as those into the PnC region indicating that the lack of arrests following neostigmine injections in the WT mice was not due the more rostral microinjection sites. Collectively, these data indicate that promoting endogenous ACh release in the pontine reticular formation of DKO, but not wild type mice increases the number, but not the duration, of narcoleptic attacks.

Finally, to determine if the neostigmine effect on DKO mice was specific to behavioral arrests, we examined the impact of these microinjections on wheel running and time spent in the inactive state. The average time the DKO mice spent in the inactive state and interacting with the running wheel is illustrated in Figure 7D and E, respectively. Neither the total time spent inactive, nor the time on the running wheel were significantly different across drug conditions based on ANOVAs (Inactivity: p = 0.08; Wheel Running: p = 0.21) although there were trends toward greater time on the wheel and less time inactive following the neostigmine microinjections. These data indicate that at the dose of neostigmine utilized, enhanced muscarinic transmission in the pontine reticular formation is sufficient to increase expression of behavioral arrests without producing a significant impact on overall levels of activity in DKO mice. These findings confirm that these regions, which are implicated in cholinergic control of REM-sleep signs in species including mouse [23], contain site(s) at which cholinergic transmission regulates the expression of rodent narcolepsy.

## Discussion

The major findings of this study are: 1) DKO mice exhibit fragmented rest states and behavioral arrests which are similar to narcolepsy symptoms observed in orexin deficient mice; 2) Low systemic doses of physostigmine and microinjections of neostigmine into the pontine reticular formation increased the number of behavioral arrests in DKO mice while having no effect upon WT mice; 3) A systemic dose of atropine decreased the number of arrests, and pontine microinjections of atropine prevented the increase in arrests induced by pontine microinjections of neostigmine; 4) The lifetimes of spontaneous behavioral arrests were exponentially distributed. Although altered cholinergic transmission changed the number of arrests, it did not change the distribution of arrest durations. As discussed below, these findings have a number of implications for both understanding the neural adaptations resulting from orexin signaling loss and for the function of the cholinergic system in regulating narcoleptic attacks.

### Are DKO mice a phenocopy of orexin ligand deficient mice?

The present observations suggest that DKO mice are a phenocopy of the orexin peptide deficient mice, at least with regard to the narcolepsy phenotype. For example, DKO mice showed evidence of an inability to maintain consolidated rest behavior compared to wild type mice (Fig. 1E, F), which is consistent with the instability in behavioral states described for ligand KO mice [27]. DKO mice also displayed unambiguous behavioral arrests that were never observed in WT mice. These arrests were similar in number, duration, variability and behavioral context to the behavioral arrests reported for orexin deficient mice. Moreover, the average number of arrests, and the time spent in arrests, increased with the time spent on the running wheel. This relation is consistent with the observation in orexin knockout mice that access to a running wheel promotes cataplexy [25]. Given that mild sleep deprivation doesn't increase cataplexy in ligand knock out mice [27], it is likely this relation relates to the enhanced motor activity or associated emotional content rather than any sleep debt accumulated during the time running. In addition to full arrests, DKO mice displayed gait disturbances and/or partial attacks which were similar in character and frequency to those reported for orexin knockout mice and appeared similar to partial cataplexy observed in human narcoleptics. In contrast, these motor alterations were never observed in OXR2 KO mice [11] underscoring a role for OX1Rs in suppressing these attributes.

Willie et al. [11] delineated two types of behavioral arrests in OX2R KO and ligand KO mice. “Abrupt” arrests interrupt ongoing active behavior and appear analogous to cataplexy, while “gradual” arrests begin from quiet waking and are more like sleep attacks. Both types of arrests were observed in ligand KO mice while abrupt arrests were rare in OX2R mice suggesting that residual signaling through OX1Rs may suppress them. While we could not systematically delineate gradual arrests using our top-mounted cameras, we observed unambiguous abrupt arrests (see supplemental videos) in DKO mice supporting a role for OX1Rs in suppressing these events. Using a side mounted camera we have also observed gradual arrests in DKO mice which began with head nodding from a stationary position (Ikinko and Leonard, unpublished observations). Willie et al. [11] found that gradual arrests in ligand KO mice, unlike those in OX2RKO mice were often longer and transitioned into a REM-like state that included rocking movements. DKO mice also showed rocking movements during behavioral arrests including those with gradual onset, further supporting a role for OX1Rs in suppressing motor aspects of these attacks.

The fraction of arrests we observed corresponding to cataplexy per se, could not be determined since our mice were not instrumented. The current consensus definition requires simultaneous recording of behavior, EEG and EMG [38] and requires at least ten seconds of immobility, an EEG dominated by theta activity, nuchal atonia and at least forty seconds of waking preceding the episode. While we only required greater than five second of immobility to define our arrests, the vast majority of arrests we observed were ten seconds or longer (see Fig. 3A) in accordance with the consensus definition. We also required only twenty seconds of purposeful behavior preceding an arrest. The consensus requirement of forty second effectively disambiguates episodes of cataplexy from direct transitions into REM sleep from waking (DREMs) which occur even in normal mice [39]. In normal mice, DREMs are often re-entries into REM sleep following a brief awakening and occur mainly in the light phase (Fujiki et al. reported only 1 of the 24 DREMs seen in normal mice occurring during the dark phase). Since our recordings were mainly conducted in the dark phase and since we never observed arrests in wild-type mice, it is unlikely that any of our scored arrests were normal DREMs erroneously scored as behavioral arrests. Moreover, requiring forty seconds of prior waking underestimates the number of cataplectic events in narcoleptic mice in the dark phase (sensitivity ∼75%) while shortening this to twenty seconds improves their detection (sensitivity ∼86%, [39]). Thus, our behavioral arrests are likely to accurately reflect both cataplexy and sleep attacks in DKO mice. Going forward, it will be important to measure EEG and EMG in conjunction with video to delineate cataplexy from sleep attacks, especially since only about 80% of abrupt arrests in ligand KO mice initiate with REM-like EEGs [11].

Collectively, our results suggest that the constitutive loss of the two known orexin receptors is sufficient to recapitulate the narcolepsy phenotype of the ligand KO and underscore the importance of OX1Rs in suppressing cataplexy and other motor signs of the phenotype.

### Altered muscarinic transmission influenced the expression of narcoleptic attacks in DKO mice

Findings with low systemic doses of physostigmine and atropine indicated that, like narcoleptic canines, muscarinic transmission regulates the expression of narcoleptic attacks in DKO mice. While the lowest two doses of physostigmine (0.01; 0.03 mg/kg) produced ∼3-fold increase in the time spent in arrests within the first hour of recording, the highest dose (0.08 mg/kg) failed to produce such an increase, and the time in arrest remained low throughout the recordings. Both peripheral and central actions of physostigmine may have contributed to this unusual dose dependence. For example, autonomic side effects such as bradycardia or tremors might have contributed to suppressing arrests at the highest dose, although signs of tremor were very rare. Centrally, perhaps cholinergic REM promoting actions predominated at low doses, while at higher doses, wake-promoting actions predominated and suppressed REM-promoting activity. This would be consistent with Datta's data indicating that moderate stimulation of PPT cholinergic regions elicits REM signs while strong stimulation elicits waking [40] with the attendant suppression of REM-sleep signs. Nevertheless, since neither wheel running nor inactivity behaviors were statistically different across physostigmine concentrations, any wake promotion by physostigmine was too subtle for our measures.

Since alterations of cholinergic transmission by the same doses of systemic physostigmine or pontine neostigmine did not produce arrests in WT mice, our data also indicate that adaptations from the loss of orexin receptor signaling leads to better coupling between cholinergic systems and the circuits that suppress muscle tone and locomotion. As noted in the Methods, the pontine neostigmine dose that increased arrests in DKO mice was more than ten times lower than a dose used to induce a REM-like state in WT mice [23] and did not produce obvious alterations in behavior in our WT control mice. This is consistent with observations in canines, where much higher doses of the cholinergic agonist carbachol delivered to the pontine tegmentum or basal forebrain are required to produce atonia in WT dogs than are required to trigger cataplexy in narcoleptic dogs [41], [42]. The specific neural changes that enhance this coupling in the absence of orexin receptor signaling are not yet clear and several mechanisms alone or in combination may contribute. For example, muscarinic transmission may be more effective at inhibiting REM-off inhibitory neurons in atonia circuits due to the absence of an opposing orexin action [43], [44]. In addition, post-synaptic sensitivity to ACh may be increased on these neurons, as suggested by receptor binding studies in the pontine reticular formation of the narcoleptic canine, which found elevated mAChRs [45]. Another possibility is that mesopontine cholinergic transmission may be enhanced in DKO mice, as appears to be the case in narcoleptic canines [46]. Consistent with this, we found higher message levels for enzymes necessary for ACh synaptic transmission in LDT samples from DKO mice [47]. These changes suggest mesopontine cholinergic transmission is enhanced in DKO mice and that inhibition of cholinesterase might have more profound consequences in DKO mice than WT mice.

### Dynamics of behavioral arrests and its modulation by cholinergic transmission

Perhaps the most striking findings of this study are: 1) that lifetimes of behavioral arrests are well described by an exponential distribution and 2) that neither systemic (see Figs. 4D, 5D) nor local (Fig. 7C) modulation of cholinergic transmission detectably changed this distribution. These observations have significant implications for the mechanisms governing the expression of narcoleptic attacks.

First, the exponential distribution suggests that the termination of spontaneous full behavioral arrests is governed by a process that is random and memory-less, like a continuous Markov process. It implies that the probability of exiting an arrest is constant per unit time and is not influenced by the number or duration of prior arrests. From this perspective, it makes sense that when the number of arrests in the first hour of recording was increased by systemic physostigmine, a few arrest bouts of longer duration would be observed (see Fig. 4). This finding is also consistent with the exponential lifetime distribution of cataplexy noted in orexin ligand knockouts [30].

A second implication relates to the role of cholinergic neurons in the expression of behavioral arrests. While the frequency of arrests was increased by both systemic physostigmine and pontine microinjections of neostigmine and was decreased by systemic atropine, the distribution of arrest durations was not altered. Thus, to the degree that cholinergic transmission was altered by our treatments, muscarinic transmission influenced transitions into behavioral arrests but played no role in stabilizing or terminating these arrests. That is, the kinetics governing transitions out of the arrest state were not influenced by muscarinic transmission since they proceeded similarly whether or not muscarinic transmission was altered. This implication places important constraints on the possible neuronal circuits regulating these arrests.

### Cholinergic enhancement of narcoleptic attacks and REM-sleep circuits

A core element of the reciprocal-interaction model is that relief from REM-off monoaminergic inhibition enables LDT/PPT cholinergic REM-on neurons to drive REM sleep signs via outputs to the pontine reticular formation (PnO, PnC and sublaterodorsal tegmental region (SLD)) and thalamus (for review see [16]). Extensive neurochemical studies of narcoleptic canines are consistent with an imbalance in this circuit promoting cataplexy. Like in narcoleptic canines, our findings in DKO mice support a role for cholinergic systems in promoting cataplexy. However, it is hard to reconcile this model with our finding that arrest lifetimes were uninfluenced by altered cholinergic transmission. In this model, REM-on cholinergic activity also excites REM-off monoaminergic neurons, critically linking cholinergic outflow to the termination of REM sleep signs. Doses of physostigmine that altered arrest frequency should also have altered arrest duration if this circuit controls behavioral arrests. Thus, our findings are inconsistent with this and other models where cholinergic outflow sets the duration of behavioral arrests. Of course, our findings do not mean that such a model might not govern normal REM sleep, our findings are just inconsistent with these circuits regulating the dynamics of behavioral arrests in narcoleptic mice. However, evidence from pontine microinjections of carbachol in normal rodents indicate that the main effect is to increase the frequency rather than the duration of REM-like events [48], [49], suggesting a similar role for cholinergic systems in generating REM signs in WT rodents.

Evidence from rodent studies has challenged the cholinergic-monoaminergic hypothesis and emphasized the role of GABAergic transmission in the SLD region [18], the ventrolateral periaquaductal grey and lateral pontine tegmentum (LPT) for REM switching [19], [20], [50]. Models derived from this work propose that reciprocating REM-on and REM-off GABAergic interconnections make up a core switch that determines the timing and duration of REM atonia [21], [22], [50]. Our findings are more consistent with this type of a model governing arrests and would be consistent with cholinergic actions at several cell groups in these models. For example, the arrangement proposed by Fuller et al. [21] in which LDT/PPT REM-on cholinergic neurons inhibit the REM-off LPT neurons but are not inhibited by the LPT would be consistent with our findings since cholinergic REM-on neurons would be outside the core switch and wouldn't determine arrest duration, yet enhanced cholinergic transmission would still promote arrests. A limitation of these models, however, is that no mechanism to terminate REM or arrest bouts are specified. One possibility consistent with the constraint implied by our findings, would be a use-dependent decay in REM-on GABAergic inhibition of REM-off GABAergic neurons. This decay would set the arrest lifetime and allow the termination of arrests to proceed independently of cholinergic influences on the LPT REM-on inhibitory neurons. Future approaches designed to selectively activate or inhibit these neurons will be necessary to provide a strong test of these ideas.

## Supporting Information

Video S1 **IR video recording of a DKO mouse illustrates the rapid onset and offset of a behavioral arrest.** After ambulation ceased, the mouse made several head movements before movement completely ceased. During the attack the mouse also made some brief rocking movements.
(MOV)

Video S2 **IR video recording of a DKO mouse whose behavioral arrest is punctuated by multiple epochs of quivering and rocking.**
(MOV)

Video S3 **IR video recording of a DKO mouse illustrates incomplete behavioral arrests that resolves without entering into a full arrest.** It appears that movement is impaired until grooming begins.
(MOV)

Video S4 **IR video recording of a DKO mouse illustrates a partial behavioral arrest that proceeded into a full arrest with considerable rocking.** Following the arrest, the mouse quickly moved to drink from the water bottle.
(MOV)
